# Assessment of Implantation With Intra-Annular Self-Expandable Device in Transcatheter Aortic Valve Replacement

**DOI:** 10.1016/j.jacasi.2026.07.011

**Published:** 2026-09-01

**Authors:** Futoshi Yamanaka, Koki Shishido, Noriaki Moriyama, Hirokazu Miyashita, Yoichi Sugiyama, Daisuke Sato, Eiji Koyama, Kunihiko Shimizu, Motoaki Kai, Takashi Matsumoto, Kazuki Tobita, Shingo Mizuno, Yutaka Tanaka, Masato Murakami, Shigeru Saito

**Affiliations:** Department of Cardiology, Shonan Kamakura General Hospital, Kamakura, Japan

**Keywords:** aortic stenosis, migration, self-expanding, TAVR

Transcatheter aortic valve replacement (TAVR) indications are expanding to younger, lower-risk patients, making long-term valve stability increasingly important.^1^ Although both balloon- and self-expandable valves (SEVs) have comparable clinical outcomes, self-expanding devices have been reported to be associated with better hemodynamic performance, especially in patients with a small aortic annulus.^2^, ^3^, ^4^ The Navitor (Abbott), an intra-annular self-expandable valve (IA-SEV), features a flexible FlexNav delivery system and NaviSeal skirt offering superior hemodynamics and reduced paravalvular leakage.^5^ Despite these beneficial features, prosthetic valve shift during final valve release could be crucial in patients with SEVs.^6^

The current study aimed to investigate the mechanisms and anatomical predictors of valve migration in patients undergoing TAVR using an IA-SEV. This retrospective study included 155 consecutive patients undergoing transfemoral TAVR with IA-SEV (May 2022-August 2024). Patients were classified into a no-migration (n = 133) and a subclinical/clinical migration (n = 22) group. Subclinical migration was defined as upward displacement of the valve frame without functional compromise (above the noncoronary cusp bottom), whereas clinical migration was defined as significant displacement requiring intervention or resulting in complications. Continuous variables were compared using Student's *t*-test or Mann-Whitney *U* test, and categorical variables using the chi-square or Fisher's exact test. Logistic regression analyses were performed using Firth's penalized likelihood method with profile likelihood-based 95% confidence intervals, using R version 4.6.0 with the *logistf* package. This study was approved by the Tokushukai Group Ethics Committee, and it was registered in the University Hospital Medical Information Network (UMIN) Clinical Trials Registry (UMIN ID: R000066237).

Baseline characteristics were comparable between the no-migration and migration groups. In the comparison of computed tomography (CT) measurements, the ascending aorta long axis 40 mm above the annulus divided by the perimeter-derived diameter (ascending aorta–annulus ratio) was significantly higher in the migration group than in the no-migration group (*P* = 0.038). Greater oversizing and left ventricular outflow tract (LVOT) calcification were more frequently observed in the migration group (*P* = 0.012 and *P* = 0.0088, respectively).

In multivariate analysis, oversizing ≥16.6% (OR: 4.61, 95% CI: 1.46-14.39; *P* = 0.010) and LVOT calcification (OR: 4.28, 95% CI: 1.16-14.83; *P* = 0.029) were independently associated with subclinical/clinical valve migration. An ascending aorta–annulus ratio of ≥1.53 was significantly associated with subclinical/clinical migration in univariate analysis. The combination of oversizing ≥16.6% and an ascending aorta–annulus ratio of ≥1.53 demonstrated high specificity (97.0%) and high negative predictive value (90.2%) for predicting subclinical/clinical migration (Figure 1).

**Figure 1:**
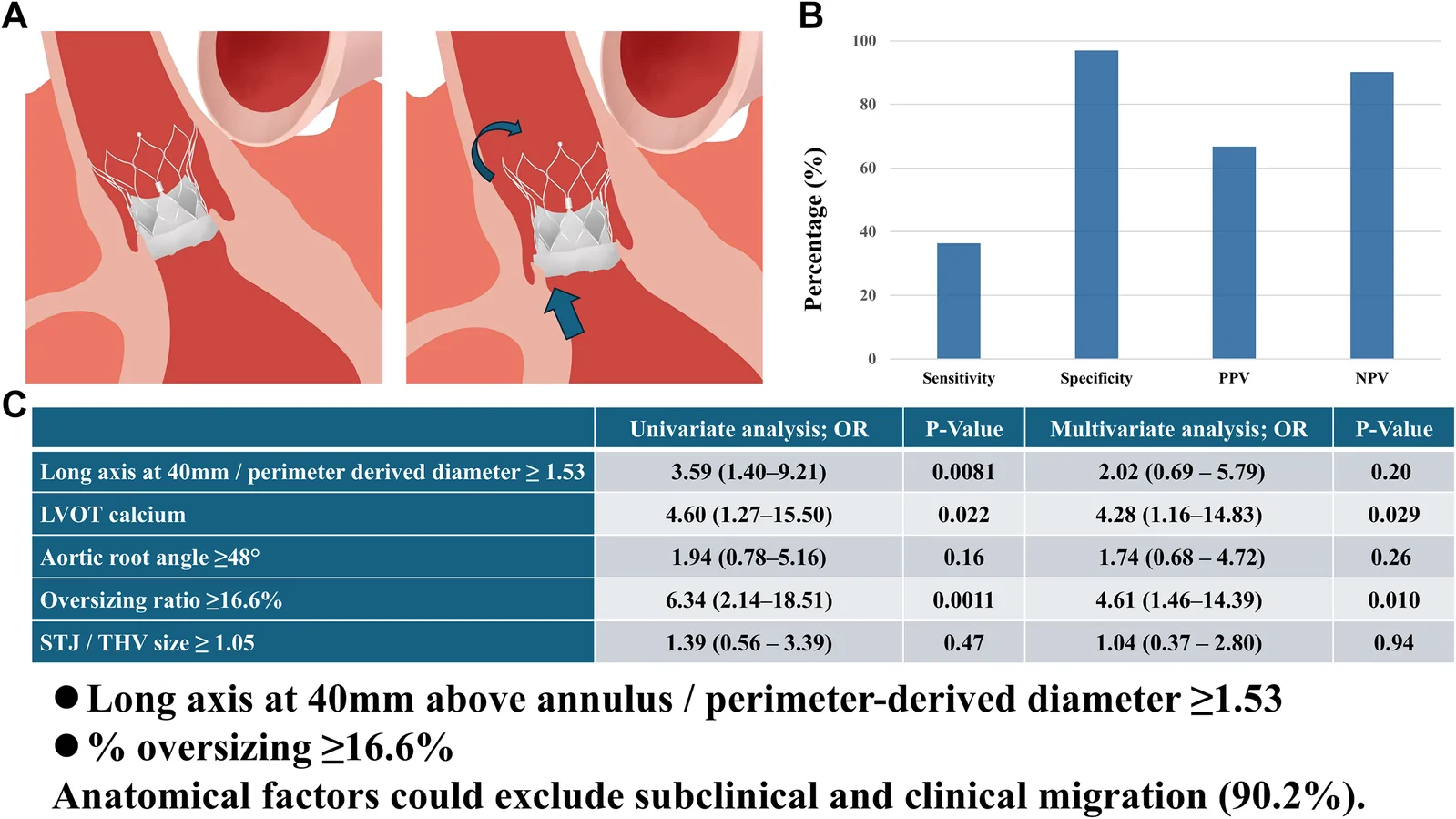
Anatomical Predictors for Subclinical and Clinical Migration (A) Illustrations demonstrate valve displacement toward the ascending aorta after deployment of an intra-annular self-expanding valve. (B) An ascending aorta–annulus ratio of ≥1.53 and excessive oversizing (≥16.6%) are associated with valve migration, with high specificity (97.0%) and high negative predictive value (90.2%) for predicting subclinical and clinical migration. (C) Multivariate analysis. LVOT = left ventricular outflow tract; NPV = negative predictive value PPV = positive predictive value; STJ/THV = sinotubular junction/transcatheter heart valve.

To our knowledge, this is the first report describing the mechanism of IA-SEV implantation, demonstrating that a greater ascending aorta–annulus ratio and oversizing ratio are significantly associated with subclinical/clinical migration. Although the intra-annular self-expandable prosthetic valve performance on echocardiographic examination at discharge was excellent, subclinical/clinical migration was observed in 14.2% of this population.

Even in cases with an unfavourable anatomy, multiple factors have an impact on valve implantation. In a particularly extreme scenario of intentionally deep implantation, besides permanent pacemaker implantation, migration toward the ascending aorta is less likely to occur. Thus, the proposed CT features do not always correspond in some cases. Valve migration has multiple causes; therefore, many clinical factors should be considered during prosthetic valve implantation.

This particular nonsupine structure contributes to greater flexibility and excellent passing ability. In contrast, a more flexible delivery system moves forward into the great curvature of the ascending aorta. Once the delivery system is released on the side of the greater curvature, the prosthetic valve is likely to shift to the side of the lesser curvature. One possible mechanism for valve migration is that a more flexible delivery system is likely to shift in exchange for excellent passing ability. To date, no reports have addressed the balance between the ascending aorta and the annulus size. Thus, we included this particular CT measurement in the multivariate analysis.

An increased oversizing ratio was independently correlated with both subclinical and clinical migration of the IA-SEV. Despite the IA-SEV having a comparable radial force, a reported limitation is a reduced opening force.^7^ The reduced opening force could be a factor resulting in underexpansion because constrained stent frames are predominantly found at the noncoronary and right coronary cusps.^8^ Once the prosthetic valve is released under expansion, caused by a weak opening force, the IA-SEV tries to open fully. Another possible mechanism of migration is that, as the oversizing ratio is increased, the valve opens more forcefully, resulting in a greater shift of motion. Predilation may play an important role in preventing this phenomenon. In this cohort, predilation was performed in all cases. More aggressive predilation using larger balloons may be crucial. IA-SEV valve deployment is influenced by anatomical features, such as the size of the ascending aorta or leaflet calcification, because of its greater flexibility and weak initial opening force. Thus, more attention should be paid to anatomical assessment using preprocedural CT.

The multivariate analysis demonstrated that an increase in oversizing ratio (%) and LVOT calcification was independently associated with subclinical and clinical migration. LVOT calcification may clinically denote a smaller pre–balloon dilation for a given annulus because of the risk of aortic root rupture. A particular case with LVOT calcification is suitable indication for SEV. This valve is characterized by better hemodynamics given a self-expandable cylindrical frame.^2^, ^3^, ^4^ A larger effective orifice area, low postprocedural mean pressure gradient, and low rate of prosthesis-patient mismatch were demonstrated in an Asian cohort study.^4^^,^^5^ Thus, we should not exclude cases with LVOT calcification.

This study has several limitations, including its retrospective design, limited sample size, absence of core laboratory CT analysis, exclusion of alternative access cases, and lack of Agatston scoring. In conclusion, the combination of an oversizing ratio ≥16.6% and ascending aorta–annulus ratio of ≥1.53 could exclude unfavorable anatomy for valve migration. A more comprehensive whole-anatomical assessment is crucial for stable implantation of IA-SEVs.

## Funding Support and Author Disclosures

The authors have reported that they have no relationships relevant to the contents of this paper to disclose.
